# Remnant Cystic Duct Carcinoma Mimicking Duodenal Carcinoma after Cholecystectomy: A Case Report

**DOI:** 10.70352/scrj.cr.26-0478

**Published:** 2026-09-12

**Authors:** Tomohiro Okura, Hideki Aoki, Hiroshi Obayashi, Eigo Oka, Hana Futagami, Maho Sato, Toshihiro Ogawa, Naoto Hori, Kou Katsuda, Kohji Tanakaya

**Affiliations:** Department of Surgery, Iwakuni Clinical Center, Iwakuni, Yamaguchi, Japan

**Keywords:** remnant cystic duct carcinoma, gallbladder carcinoma, cholecystectomy, duodenal obstruction

## Abstract

**INTRODUCTION:**

Cystic duct carcinoma is an uncommon subtype of gallbladder cancer, and remnant cystic duct (RCD) carcinoma after cholecystectomy is particularly rare. Due to the rarity of this disease and the anatomical alterations following cholecystectomy, early diagnosis can be challenging, making it essential to consider this entity in the differential diagnosis. We report a rare case of RCD carcinoma that became clinically apparent 3 years after laparoscopic cholecystectomy for acute cholecystitis, presenting with duodenal obstruction and initially suspected to be duodenal cancer.

**CASE PRESENTATION:**

A 53-year-old man had undergone laparoscopic cholecystectomy for acute cholecystitis with gallstones 3 years earlier at another hospital. He presented to our institution with weight loss, loss of appetite, and vomiting. Endoscopic biopsy was inconclusive; therefore, we performed open surgery to obtain a duodenal biopsy, which revealed adenocarcinoma, followed by a gastrojejunostomy. Two months later, we performed subtotal stomach-preserving pancreaticoduodenectomy, partial hepatectomy, and partial transverse colectomy. The final pathological diagnosis was RCD carcinoma with invasion into the duodenum, liver, and transverse colon. The patient was discharged on POD 24. He has remained disease-free during the first postoperative year.

**CONCLUSIONS:**

We report a rare case of RCD carcinoma after cholecystectomy. This case underscores the importance of considering RCD carcinoma as a potential biliary tract malignancy in patients presenting with unexplained symptoms following cholecystectomy.

## Abbreviations

AJCC
: American Joint Committee on Cancer
ALT
: alanine aminotransferase
AST
: aspartate aminotransferase
CA19-9
: carbohydrate antigen19-9
CD
: cystic duct
CEA
: carcinoembryonic antigen
CK7
: cytokeratin7
CK20
: cytokeratin20
FDG
: fluorodeoxyglucose
MRCP
: magnetic resonance cholangiopancreatography
RCD
: remnant cystic duct
SSPPD
: subtotal stomach-preserving pancreaticoduodenectomy
UICC
: Union Internationalis Contra Cancrum

## INTRODUCTION

CD cancer was first included in the staging classification scheme for gallbladder cancer in the 7th edition of the AJCC Cancer Staging Manual published in 2010, and it is considered an extremely rare malignancy of the biliary tract.^1,2)^ Carcinoma arising from the RCD is even more uncommon. To the best of our knowledge, only 11 cases of RCD carcinoma have been reported in the English literature since the first description by Kuwayti et al. in 1957.^3)^ Due to the rarity of this disease and the anatomical alterations following cholecystectomy, early diagnosis can be challenging. Here, we report a rare case of RCD carcinoma that became symptomatic 3 years after laparoscopic cholecystectomy, presenting with duodenal obstruction and initially suspected to be primary duodenal carcinoma.

## CASE PRESENTATION

**Fig. 1:**
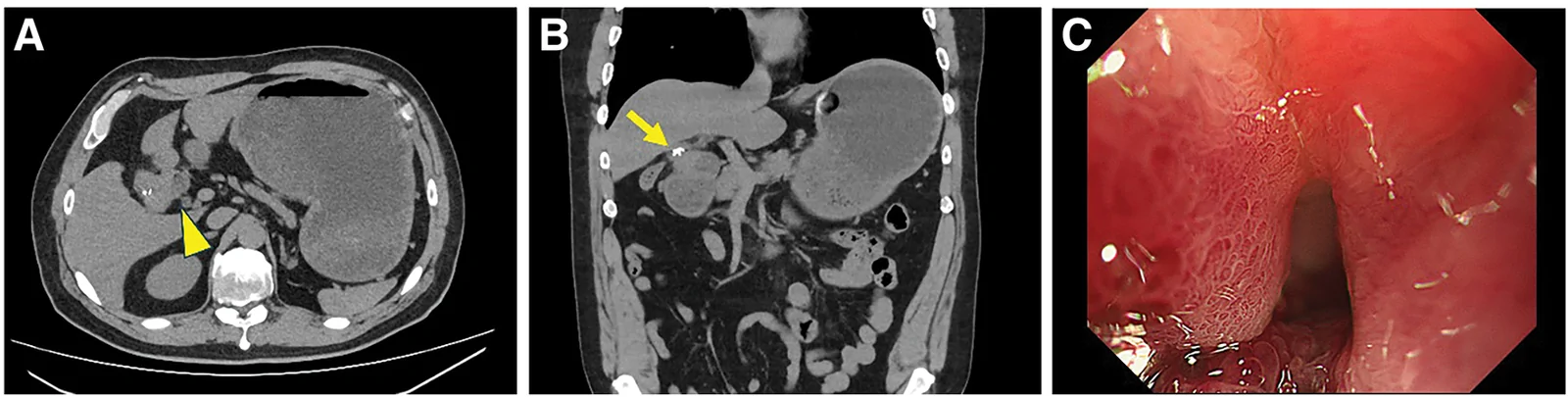
Contrast-enhanced abdominal CT and upper endoscopy images at the first admission. (**A**) The yellow arrowhead indicates the stenosis in the duodenal bulb. (**B**) The yellow arrow points to the clip placed on the CD during the prior cholecystectomy. (**C**) Endoscopic findings showing circumferential narrowing of the duodenum, although no obvious mucosal irregularity is observed. CD, cystic duct

A 53-year-old man presented to our emergency department with a 2-month history of loss of appetite and nausea, and was admitted with a diagnosis of intestinal obstruction due to a stricture of the duodenal bulb (**Fig. 1A**, **1B**). Upper endoscopy revealed severe stenosis in the duodenal bulb; the endoscope could not be advanced beyond the stenotic segment, and no obvious neoplastic changes were observed on the mucosal surface within the visible field (**Fig. 1C**). After 2 weeks of conservative treatment failed to improve the obstruction, an open gastrojejunal bypass procedure was performed. A biopsy taken from the outer wall of the duodenum revealed adenocarcinoma, and he was diagnosed with duodenal cancer.

His past medical history included hypertension and type 2 diabetes mellitus. Three years earlier, he had undergone laparoscopic cholecystectomy for acute cholecystitis due to gallbladder stones at another hospital. Macroscopic examination of the resected gallbladder revealed marked thickening of the gallbladder wall, consistent with chronic cholecystitis. Two gallstones were identified within the gallbladder, and no gross tumorous lesions were observed. Histopathological examination revealed acute cholecystitis with extensive erosion of the gallbladder mucosa. No cytological epithelial atypia was identified. Marked inflammatory cell infiltration composed of lymphocytes, plasma cells, and eosinophils was observed. No evidence of malignancy was identified at the CD resection margin.

**Fig. 2:**
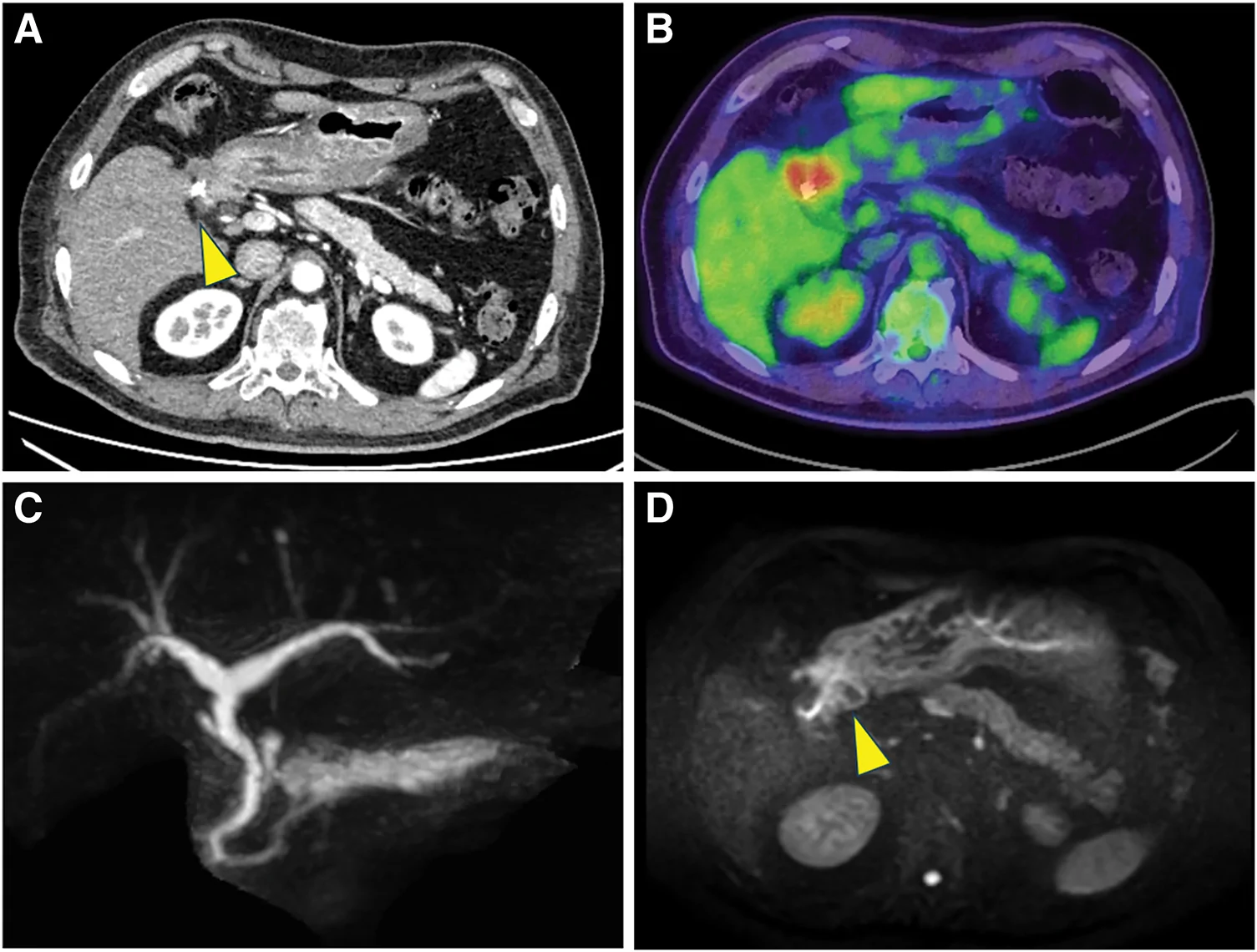
Contrast-enhanced abdominal CT, ^18^F-FDG PET images, and MRCP obtained before SSPPD. (**A**) The arrowhead indicates the area where the tumor is invading the liver. (**B**) ^18^F-FDG PET shows abnormal FDG uptake corresponding to the lesion (SUVmax: 6.757). (**C**) There is no dilation of the common bile duct or the main pancreatic duct. (**D**) The arrowhead indicates diffusion restriction in the duodenal wall. FDG, fluorodeoxyglucose; MRCP, magnetic resonance cholangiopancreatography; SSPPD, subtotal stomach-preserving pancreaticoduodenectomy

Blood tests showed that the tumor markers CEA and CA19-9 were within normal ranges. At the initial presentation, AST and ALT levels were mildly elevated (41 U/L and 59 U/L) but normalized after the gastrojejunal bypass procedure. In contrast, cholestatic enzymes and serum bilirubin levels remained within the normal range throughout the clinical course. Contrast-enhanced abdominal CT suggested that the duodenal tumor had invaded the liver and transverse colon (**Fig. 2A**). Fluorine-18 FDG-PET demonstrated FDG accumulation in the duodenal tumor, with a maximum standardized uptake value of 6.757 (**Fig. 2B**). MRCP revealed no dilatation of the common bile duct or the main pancreatic duct (**Fig. 2C**). Although diffusion restriction was observed in the duodenal wall, no definite mass lesion was identified (**Fig. 2D**). No findings suggestive of distant metastasis were observed. No pancreaticobiliary maljunction was identified. We planned to perform pancreaticoduodenectomy with lymph node dissection following 2 cycles of neoadjuvant FOLFOX chemotherapy.

**Fig. 3:**
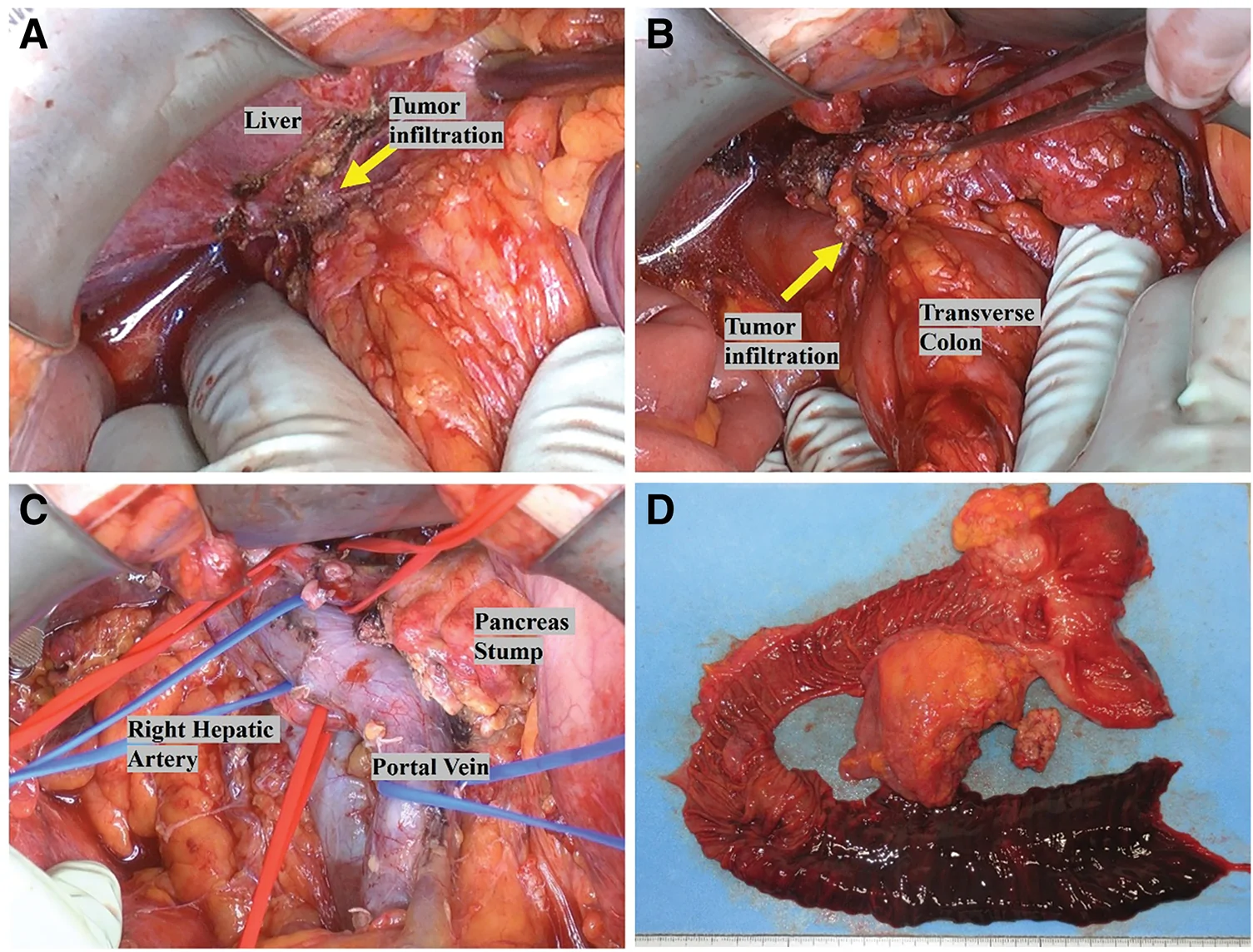
Intraoperative view and the excised specimen. (**A**) The yellow arrow indicates tumor invasion into the liver. (**B**) The yellow arrow indicates tumor invasion into the transverse colon. (**C**) Intra-abdominal view after completion of the subtotal stomach-preserving pancreaticoduodenectomy. (**D**) The resected specimen demonstrates a tumor located within the duodenal wall.

**Fig. 4:**
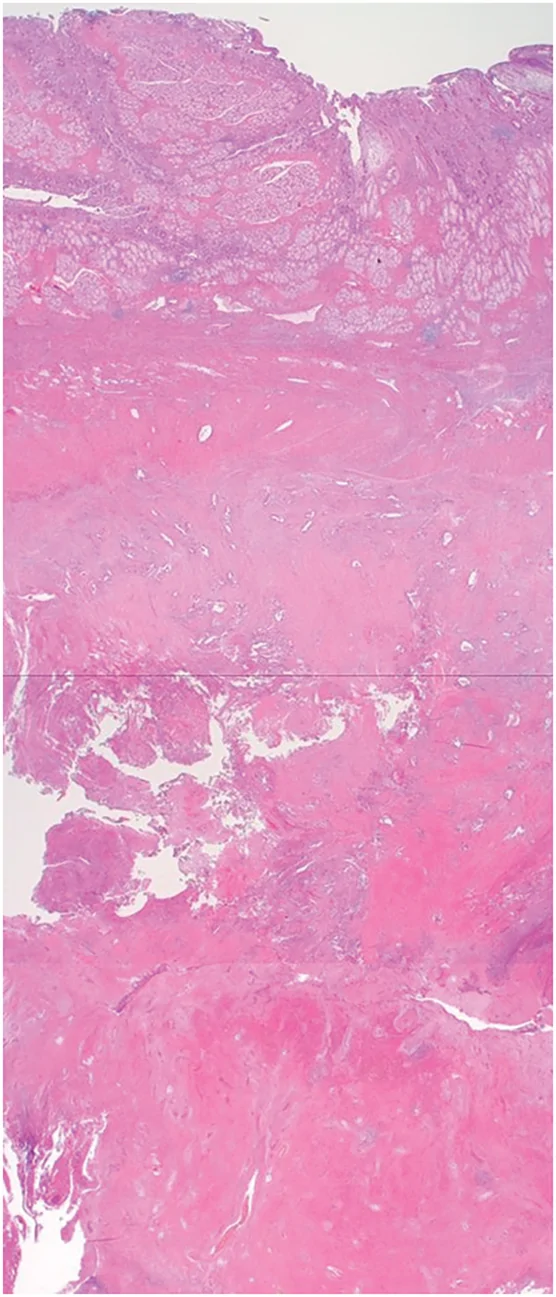
H&E-stained section of the duodenum showing the mucosa to deeper layers. No tumor cells are present on the mucosal surface; tumor cell infiltration is confined to the submucosa and deeper layers.

**Fig. 5:**
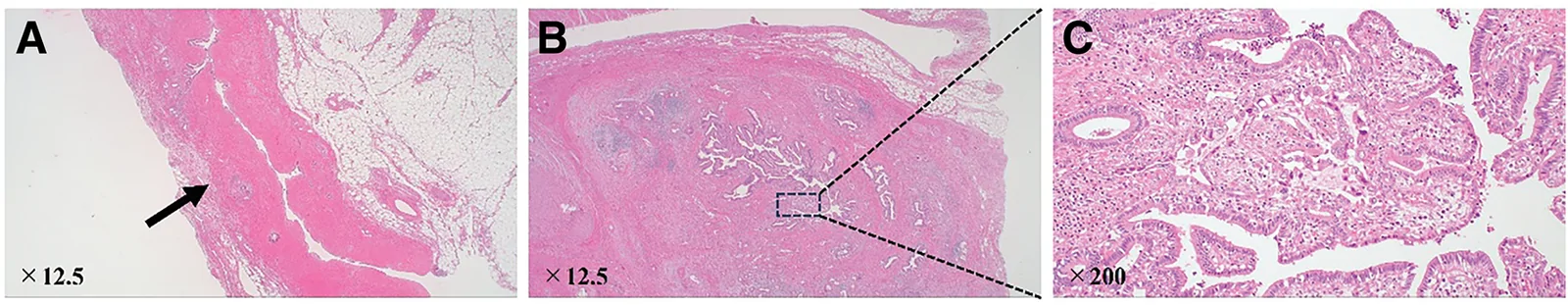
H&E-stained sections of the extrahepatic bile duct and the RCD. (**A**) No tumor cells were detected in the extrahepatic bile ducts (×12.5). (**B**, **C**) Tumor cells were identified within the epithelium of the RCD stump with infiltration into the surrounding tissue (**B**: ×12.5; **C**: ×200). RCD, remnant cystic duct

**Fig. 6:**
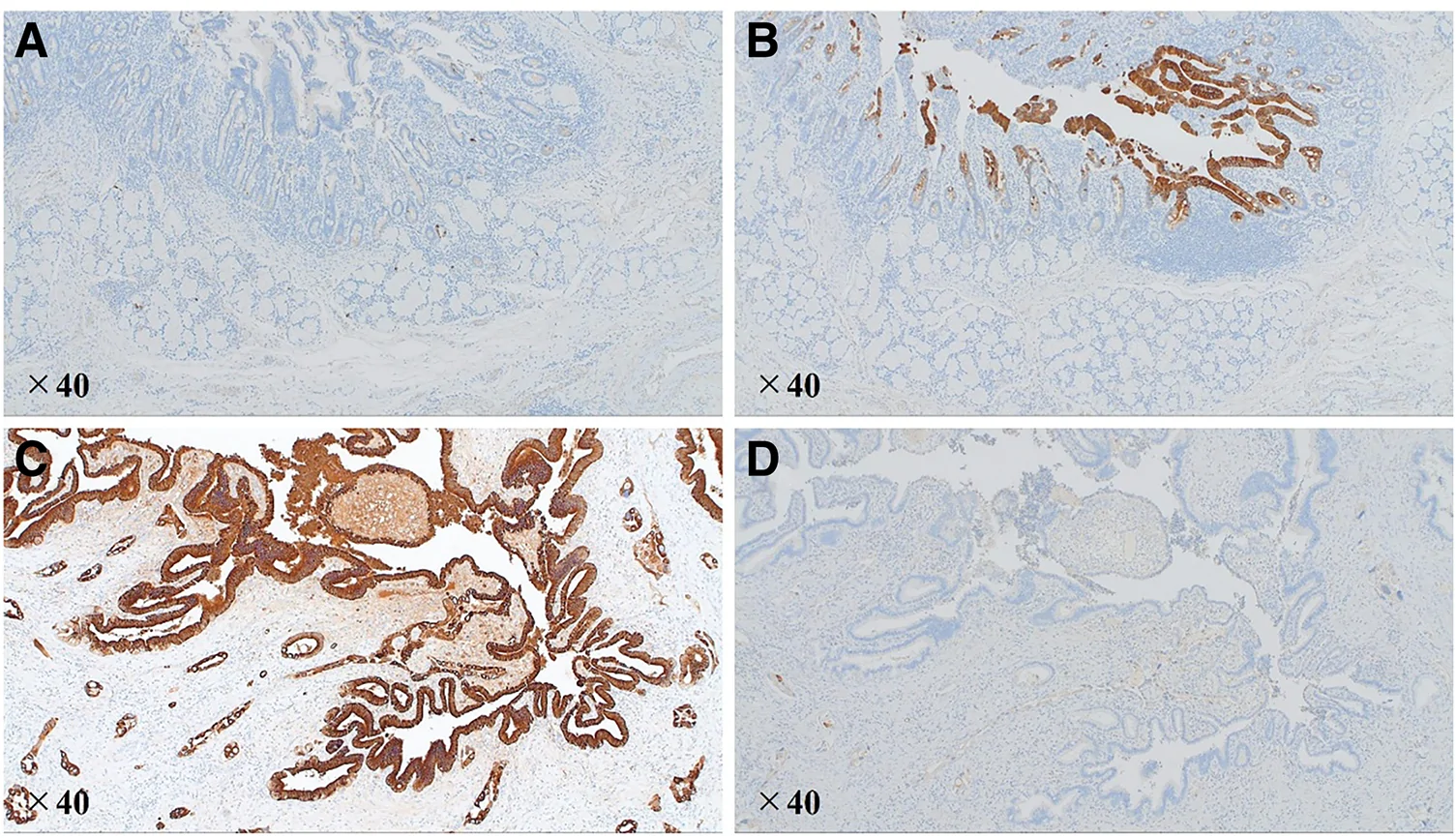
Immunohistochemically stained sections of the duodenal mucosa and epithelium of the RCD. (**A**, **B**) The duodenal mucosa was negative for CK7 and partially positive for CK20 (×40). (**C**, **D**) The epithelium of the RCD was positive for CK7 and negative for CK20 (×40). CK7, Cytokeratin7; CK20, Cytokeratin20; RCD, remnant cystic duct

Under general and epidural anesthesia, an upper midline abdominal incision was made. A tumor was palpated at the superior duodenal angle, with invasion into the liver bed and the transverse colon. Therefore, partial liver resection and resection of the involved transverse colon wall were performed (**Fig. 3A**, **3B**). Subsequently, SSPPD with lymph node dissection and modified Child reconstruction was carried out (**Fig. 3C**, **3D**). The resected specimen demonstrated a tumor located within the duodenal wall; however, no exposure of the tumor on the mucosal surface was identified. The operative time was 10 hours and 41 minutes, and blood loss was 953 mL. No blood transfusion was required. Histopathological examination revealed the absence of tumor cells on the duodenal mucosal surface, whereas extensive tumor infiltration was observed from outside the duodenal wall into the submucosal layers (**Fig. 4**). No tumor cells were detected in the extrahepatic bile ducts (**Fig. 5A**). In contrast, tumor cells were identified in the CD epithelium of the resected specimen and showed extensive invasion into the surrounding tissues (**Fig. 5B**, **5C**). Immunohistochemical staining demonstrated that the duodenal mucosa was negative for CK7 and partially positive for CK20, whereas the RCD epithelium was positive for CK7 and negative for CK20 (**Fig. 6A**–**6D**). Based on these findings, the final diagnosis was RCD carcinoma. Tumor cells were found to have infiltrated the resected liver tissue and the wall of the transverse colon, but no infiltration was observed at any of the resection margins. There was also no evidence of lymph node metastasis. According to the eighth edition of the AJCC/UICC staging system, the tumor was classified as pT4N0M0, Stage IVA. Postoperatively, the patient developed biochemical leakage-related pancreatic fistula and cholangitis, but he was discharged home on POD 24. He received 6 months of adjuvant oral S-1 therapy and has remained disease-free during the first postoperative year.

## DISCUSSION

CD carcinoma was first reported by Farrar in 1951 and is considered a relatively rare malignant tumor of the biliary tract.^4)^ Farrar defined CD carcinoma based on the following 3 criteria: “(1) The growth must be restricted to the CD; (2) There must be no neoplastic process in the gallbladder, hepatic or common bile ducts; and (3) A histological examination of the growth must confirm the presence of carcinoma cells”.^4)^ In 2003, Ozden et al. proposed diagnostic criteria stating that, even in tumors for which the primary site cannot be clearly determined among the gallbladder, common bile duct, and CD, lesions whose epicenter is located in the CD should be classified as CD carcinoma.^5)^ Yu et al. suggested that CD carcinoma should be classified as an extrahepatic cholangiocarcinoma rather than a gallbladder cancer, and many aspects of its clinical and pathological characteristics remain poorly understood.^6)^ In the present case, the patient initially presented with symptoms of duodenal stenosis and was diagnosed with duodenal cancer after adenocarcinoma was detected in a biopsy obtained from the duodenal wall. However, postoperative pathological examination revealed that the tumor originated in the RCD, leading to a final diagnosis of RCD carcinoma.

Since the first report by Kuwayti et al. in 1957, only 11 cases of RCD carcinoma have been reported in the English literature, making it an exceptionally rare malignancy.^3,7–16)^ Including our case, 3 were from the United States and 9 were from Asia. The median age was 66 years, and the cohort consisted of 9 male and 3 female patients. The primary indications for the initial cholecystectomy were acute cholecystitis in 5 cases, gallstone disease in 5 cases, and chronic cholecystitis in 2 cases. The median interval between cholecystectomy and the onset of RCD carcinoma was 5.5 years. Among the 12 cases, several were highly advanced, with invasion into the stomach, duodenum, transverse colon, or right hepatic artery, and 1 case developed liver metastasis 8 months after surgery. Because of the anatomical location of the CD, CD carcinoma is more likely to cause jaundice; consequently, some reports suggest that symptoms tend to appear at an earlier stage than in gallbladder cancer.^13,17)^ However, in cases following cholecystectomy, there is a possibility of anatomical variations resulting from the disruption of the CD’s structural integrity or postoperative adhesions. Therefore, early diagnosis is not always achieved, and some cases are diagnosed only after invasion into other organs has occurred. Among the 11 previously reported cases, only 2 cases were diagnosed as RCD carcinoma due to obstructive jaundice. The remaining cases included 2 cases with duodenal invasion, 1 case with transverse colon invasion, and 1 case with gastric invasion. Even in advanced cases, biliary obstruction or obstructive jaundice does not necessarily occur. Therefore, careful evaluation is required in the absence of these findings. In this case, it was considered that the duodenum had adhered to the area surrounding the CD stump as a result of laparoscopic cholecystectomy for acute cholecystitis, leading to tumor infiltration of the duodenum and the onset of symptoms due to duodenal stricture. Endoscopy revealed no obvious epithelial atypia on the duodenal mucosa. Because the tumor lesion was identified around the clip used for CD stump, RCD carcinoma should have been considered in the differential diagnosis from the initial presentation. However, given the patient's markedly compromised general condition, immediate radical surgery was considered to carry a high risk of perioperative complications. Therefore, the planned 2-stage curative resection was selected.

During cholecystectomy, if the gallbladder is severely inflamed, a subtotal cholecystectomy may be required, or it may not be possible to transect the CD at the junction of the CD, common hepatic duct, and common bile duct. In this case, although the CD had been resected during a cholecystectomy performed at another hospital, the cancer was believed to have arisen from the RCD that had been clipped. Although extremely rare, it is important to recognize that carcinoma can develop in the RCD following cholecystectomy.

## CONCLUSIONS

We reported an extremely rare case of RCD carcinoma that presented with duodenal obstruction and was initially suspected to be primary duodenal cancer. Because the CD had been clipped during a prior cholecystectomy and postoperative anatomical alterations were present, an accurate preoperative diagnosis could not be achieved. RCD carcinoma may present with symptoms such as obstructive jaundice or gastrointestinal obstruction and should be considered in the differential diagnosis of malignancies developing after cholecystectomy.

## DECLARATIONS

### Availability of data and materials

Data sharing is not applicable to this article.
